# Construction workers’ depression, anxiety, stress, and risk factors in China: a cross-sectional study

**DOI:** 10.7189/jogh.15.04167

**Published:** 2025-07-21

**Authors:** Zhicheng Ling, Yuying Xu, Minmin Tao, Binbin Zhang, Meng Zhang, Zhiding Zhang, Xiaoya DA, Xinmin Liu, Long Huang

**Affiliations:** 1 College of Humanities and Management, Wannan Medical College, Wuhu, China; 2 Department of Anesthesiology, Wannan Medical College, Wuhu, China; 3 Clinical Medical College, Wannan Medical College, Wuhu, China; 4 School of Laboratory Medicine, Wannan Medical College, Wuhu, China

## Abstract

**Background:**

Frontline construction workers are generally faced with risk factors such as alcoholism, smoking, and being far away from home, which pose a great threat to their mental health. However, this issue has not yet attracted significant attention form the global community. For this reason, we examined depression, anxiety, and stress levels among construction workers in China and identified their key risk factors, such as education, occupational tenure, geographical mobility, physical well-being, COVID-19 status, insomnia, and alcohol dependency.

**Methods:**

We conducted an online survey using validated scales, including the Depression, Anxiety, and Stress Scale, Insomnia Severity Index Scale, Alcohol Dependence Scale, Family-work Conflict Scale, Leadership Support Scale, Workplace Exclusion Scale, and Proactive Personality Scale.

**Results:**

We analysed 912 valid responses (790 males, 122 females; mean age = 36.35 years (standard deviation = 10.11). Depression, anxiety, and stress levels were significantly influenced by age, education, work-related injuries, COVID-19 status, insomnia, alcohol dependence, workplace exclusion, and work-family conflict among construction workers (all *P*-values < 0.05). The regression analysis showed that work-family conflict, workplace exclusion, alcohol dependence, and insomnia were positively associated with depression (*P* < 0.001), while proactive personality and leadership support were negatively associated with depression (all *P*-values <0.05). Similarly, physical health, workplace exclusion, alcohol dependence, and insomnia were positively associated with anxiety (all *P*-values <0.001). Additionally, having a proactive personality negatively influenced depression (*P* < 0.001). Anxiety positively predicted physical health issues (*P* < 0.001), workplace exclusion (*P* < 0.001), alcohol dependence (*P* < 0.001), and insomnia (*P* < 0.001), whereas leadership support reduced anxiety levels (*P* = 0.01).

**Conclusions:**

Mental health risks among construction workers are linked to work and personal factors, including insomnia, alcohol dependence, workplace exclusion, and work-family conflict. Employers should implement targeted interventions to improve the work environment, leadership support, and social support systems to enhance the workers’ mental well-being.

The mental health of construction workers substantially contributes to productivity loss and safety concerns. Previous research has shown that occupational stress is a significant mental health challenge that leads to serious productivity- and safety-related issues [1,2]. From a social and environmental perspective, construction workers are impacted to a great extent by outdated production management models, hazardous work environments, and high-pressure job conditions, alongside other factors. Williams [3] developed a stress-injury causality model that explains how organisational stress stemming from job demands and inadequate supervisor support and training can directly or indirectly increase the occurrence of accidents, near misses, and injuries. Similarly, Campbell and colleagues [4] found that several stressors, including environmental, organisational, and work-related factors, are strongly associated with safety and productivity performance. Bowen and colleagues [5] similarly identified various organisational stressors that contribute to accidents, such as unfair rewards, inappropriate use of safety equipment, lack of goal setting, and an unsafe physical work environment. Such occupational pressures not only impair individual job performance, but also hinder teamwork and coordination, ultimately exacerbating safety and productivity issues at construction sites [6–8].

From a personal perspective, the main factors affecting construction workers include personal temperament, physical and mental illnesses, family environment, and age structure [9]. Personal temperament can control employees' risk-taking behaviours and make them behave safely or unsafely [10]. Liang and colleagues [11] found that construction workers with aggression, impatience, and inability to relax behaviour patterns were more susceptible to stress, leading them to engage in risky behaviours. Research evidence supports that extroversion, openness, agreeableness, and rigour influenced self-perceived fatigue and safety culture on construction sites [12]. Mood disorders such as depression and anxiety have a crucial impact on productivity and safety [13]. Siu and colleagues [14] found that psychological distress, including depression and anxiety, is positively correlated with the number of accidents and occupational injuries. Haslam and colleagues [15] explained that depression and anxiety in workers may cause inattention, emotional distress, motivation, and decision-making difficulties. In fact, depression has been identified as the most serious mental health problem among bricklayers and site supervisors. For example, studies exploring the risk factors for workers’ mental health found that those over 60 years of age have relatively poor levels in the four dimensions of terror, somatisation, paranoia, and obsessive-compulsive symptoms [16].

In terms of cultural and educational levels, significant differences were found in the three dimensions of depression, anxiety, and paranoia, with the scores of junior high school and below and high school level students in China having higher values than those of college education level or above [17]. This suggests that people with higher literacy levels are less likely to develop mental health symptoms [18]. Individual studies have also explored factors that influence the mental health of construction workers and have found that two-fifths of them suffer from depression and experience trait anxiety [19]. More concerningly, three-fifths of workers reported alcohol-related problems.

Mental health is therefore related to many demographic factors and is substantially influenced by sleep quality and family-work conflict [20]. However, few studies have specifically examined construction workers as a distinct group, and none have focussed on their mental health status and risk factors post-pandemic. With this in mind, we explored the anxiety, depression, and stress levels among Chinese construction workers and the influence of demographics, insomnia, and work-family conflicts thereon.

## METHODS

### Study sample

We surveyed 1000 employees of the China Communications Construction Second Aviation Engineering Bureau through the WJX.cn online questionnaire system in cooperation from April to May 2023. We excluded questionnaires with incomplete, too short or long, and irregular answers (repetitive, identical responses to all questions), retaining 912 valid responses from 790 males and 122 females with an average age of 36.35 years (standard deviation = 10.11).

### Questionnaire structure

#### Sociodemographic data

We collected the following sociodemographic data: gender, age, marital status (single, married), education level (high school and below, college, undergraduate, and above), position (managers, employees), and whether employees and their spouses worked in different places (yes, no) were collected.

#### Depression, Anxiety, and Stress Scale

We used the Depression, Anxiety, and Stress Scale (DASS-21) to assess psychological distress. This widely-used scale comprises 21 items divided into three subscales: depression, anxiety, and stress, each containing seven items [21]. Participants rated each item on a four-point Likert scale ranging from 0 (completely unqualified) to 3 (completely qualified). For example, one item on the scale is: ‘I find myself increasingly restless.’ To obtain the subscale scores, we summed the scores of all items within each subscale and multiplied them by 2. The Cronbach's α coefficients for the depression, anxiety, and stress subscales in our study were 0.829, 0.875, and 0.922, respectively, indicating strong internal consistency reliability for each subscale.

#### Insomnia Severity Index Scale

The Insomnia Severity Index (ISI) is a standardised instrument designed to assess the variety and intensity of three core insomnia symptoms: sleep satisfaction, the perceived significance of others' sleep disturbances, and the impact of sleep-related issues on daily functioning [22]. It uses a Likert scale scoring system ranging from 0 (absent) to 4 (extremely severe). The cumulative score ranges from 0 to 28, with the following categories: no insomnia (0–7), mild insomnia (8–14), moderate insomnia (15–21), and severe insomnia (22–28). The ISI demonstrates robust reliability and validity across general and clinical populations [21], with a Cronbach's α coefficient of 0.904 observed in our study.

#### Alcohol Dependence Scale

The Alcohol Dependence Scale measures the severity of alcohol dependence. The scale has 25 items scored on a four-point Likert scale (range: 0–3). The total score ranges from 0 to 47 and can be divided into no insomnia (0), mild (1–13), moderate (14–21), severe (22–30), and severe alcohol dependence (31–47), according to the score. The Cronbach's α coefficient of this scale for our study was 0.895.

#### Family-work Conflict Scale

We used the Family Work Conflict Scale developed by Netemeyer [23], which comprises five items that assess the extent of role interference between the family and occupational domains. One example item from this scale would be ‘The demands of family or spouse/partner interfere with my work activities’. Higher composite scores indicate greater severity of family-to-work conflict. This subscale demonstrated high internal consistency, with a Cronbach's alpha coefficient of 0.926.

#### Leadership Support Scale

We used the Leadership Support Scale, a four-item scale adapted from Cheng and colleagues’ validated instrument, designed to assess perceived supervisory support during workplace adversity [24]. One example item from this scale is ‘When I encounter difficulties or crises, my supervisor provides assistance.’ The total score is obtained by summing the responses for all items, with higher values reflecting stronger perceptions of leadership support. The scale had high internal consistency in our study, with a Cronbach's α coefficient of 0.929.

#### Workplace Exclusion Scale

The Workplace Exclusion Scale is a psychometrically validated 10-item scale that captures multidimensional exclusion experiences across the cognitive, affective, and behavioural domains. Participants rated items (*e.g.* ‘Colleagues deliberately avoided inviting me to work-related discussions’) on a five-point Likert scale (1 = strongly disagree to 5 = strongly agree). We derived composite scores through either summative aggregation (theoretical range: 10–50) or dimensional mean calculations for the subconstruct analysis. The scale also had high reliability in our study, with a Cronbach's α of 0.913.

#### Proactive personality scale

The 17-item Proactive Personality Scale was developed and translated from English to Chinese by Zhang and colleagues [25] based on the work of Bateman and Crant [26]. It measures how individuals exhibit proactive behaviours and attitudes in various contexts. It is designed as a seven-point Likert scale, with scores ranging from 1 to 7, *i.e.* from ‘completely disagree’ to ‘completely agree’. Among them, four items require reverse scoring, meaning that a response of ‘1’ would be reverse-scored as ‘7’, for example. The average or total score of a scale is calculated by adding up the scores of all items and then dividing by 17 to obtain the average score. Alternatively, the total score can also be calculated by directly adding up the scores of all items. The maximum score is 119 points. Usually, a score of 10–30 can be considered as indicative of a low-level proactive personality, 31–50 as indicative of a moderate level proactive personality, and 51–70 as indicative of a high-level proactive personality. The total score is determined by calculating the average or total scores, with 14 items being reverse-scored. The Cronbach's α coefficient for this scale was 0.854, indicating high internal consistency reliability.

### Discrimination analysis

According to the discrimination test results (**Table 1**), there were statistically significant differences in alcohol dependence, insomnia index, work-family conflict, leadership support, workplace exclusion, proactive personality, total stress score, total anxiety score, and total depression score between the stratified groups (*P* < 0.001). The significance level rejects the null hypothesis, indicating that the scale items of each variable are highly differentiated, which means that the design is reasonable. This indicates the robustness of the scales used in this study, as they effectively distinguished the examined structures at different levels in the sample population.

**Table 1:** Discrimination analysis

	Group, mean (SD)			
	**0–27%**	**27–73%**	**73–100%**	***t***
**Alcohol dependence**	0.19 (0.40)	5.13 (2.74)	17.00 (6.45)	−13.51
**Insomnia index**	2.30 (1.20)	7.98 (1.80)	15.44 (3.86)	−16.91
**Work and family conflict**	2.16 (0.83)	4.83 (0.73)	6.40 (0.41)	−23.76
**Leadership support**	3.53 (0.79)	5.27 (0.53)	6.55 (0.47)	−17.03
**Workplace exclusion**	1.20 (0.18)	2.13 (0.22)	3.08 (0.50)	−18.36
**Proactive personality**	3.34 (0.76)	4.82 (0.46)	6.26 (0.47)	−16.95
**Stress**	0.37 (0.79)	6.70 (3.27)	19.41 (7.40)	−13.30
**Anxiety**	0.37 (0.79)	4.65 (2.39)	15.85 (7.76)	−10.31
**Depression**	0.00 (0.00)	3.39 (2.60)	16.67 (8.41)	−10.30

### Statistical analysis

We used SPSS, version 24.0 (IBM Corp., Armonk, New York, USA) for analysis. We reported our data as means and standard deviations (for continuous variables) or as frequencies and percentages (categorical variables). We used the χ^2^ test to compare the the differences between different levels. We further performed logistic regression analysis, using the correlation coefficient and its 95% confidence interval (CI) to estimate the influencing factors of SHS. We set the statistical significance threshold at *P* < 0.05.

### Ethics

All subjects who participated in the survey signed an informed consent form. The survey has received approval from the Academic Ethics Committee of Wannan Medical College (2024-024).

## RESULTS

### General demographic characteristics

Age significantly correlated with depression, anxiety, and stress levels, with individuals aged <35 years having higher rates of depression, anxiety, and stress compared to those aged ≥35 years (all *P*-values <0.05). The χ^2^ values from the correlation tests were all <0.05, with corresponding *P*-values also being <0.05, indicating a significant impact of age on anxiety, depression, and stress levels. Although the rates of depression and anxiety were slightly higher for individuals in urban settings than those in non-urban settings, the difference was not statistically significant. However, there was a significant difference in stress levels, with individuals in urban settings experiencing significantly lower stress levels than those in non-urban settings (*P* = 0.027), suggesting a possible association between urban settings and lower stress levels. Individuals with shorter work tenure (<5 years) had significantly higher rates of depression, anxiety, and stress than those with longer work tenure (≥5 years), indicating a significant impact of work tenure on levels of anxiety, depression, and stress. Factors such as workplace injury, working in different locations, physical health status, COVID-19 infection status, severity of COVID-19 impact, insomnia, and alcohol dependency significantly influenced depression, anxiety, and stress rates. The levels of depression, anxiety, and stress of individuals with high education were significantly higher than those of low education groups (all *P*-values <0.05). Work-related injuries positively predicted depression, anxiety, and stress levels (all *P*-values <0.05). Patients with COVID-19 had more significant anxiety, and those with a more severe influence of COVID-19 had a significantly higher risk of anxiety (*P* = 0.037). Workers with insomnia had higher levels of depression (*P* < 0.001), anxiety (*P* < 0.001), and stress (*P* < 0.001) than those without insomnia. Workers with alcohol dependence had higher levels of depression (*P* < 0.001), anxiety (*P* < 0.001), and stress (*P* < 0.001) than those without alcohol dependence (**Table 2**).

**Table 2:** Prevalence of depression, anxiety, and stress by sociodemographic characteristics

		Depression, n (%)				Anxiety, n (%)				Stress, n (%)			
	**n**	**No**	**Yes**	**χ^2^**	***P*-value**	**No**	**Yes**	**χ^2^**	***P*-value**	**No**	**Yes**	**χ^2^**	***P*-value**
**Gender**				0.014	0.906			0.355	0.552			0.045	0.832
Male	790	561 (71.01)	229 (28.99)			514 (65.06)	276 (34.94)			641 (81.14)	149 (18.86)		
Female	122	86 (70.49)	36 (29.51)			76 (62.30)	46 (37.70)			98 (80.33)	24 (19.67)		
**Age**				6.89	0.009			5.105	0.024			11.315	0.001
<35 years	475	319 (67.16)	156 (32.84)			291 (61.26)	184 (38.74)			365 (76.84)	110 (23.16)		
≥35 years	437	328 (75.06)	109 (24.94)			299 (68.42)	138 (31.58)			374 (85.58)	63 (14.42)		
**Same-city residence**				9.997	0.007			3.31	0.191			7.22	0.027
Yes	260	204 (78.46)	56 (21.54)			180 (69.23)	80 (30.77)			225 (86.54)	35 (13.46)		
No	458	312 (68.12)	146 (31.88)			289 (63.10)	169 (36.90)			362 (79.04)	96 (20.96)		
Single	194	131 (67.53)	63 (32.47)			121 (62.37)	73 (37.63)			152 (78.35)	42 (21.65)		
**Educational attainment**				9.885	0.007			10.851	0.004			7.928	0.019
High school or below	82	69 (84.15)	13 (15.85)			63 (76.83)	19 (23.17)			72 (87.80)	10 (12.20)		
Associate degree	111	84 (75.68)	27 (24.32)			81 (72.97)	30 (27.03)			98 (88.29)	13 (11.71)		
Bachelor's degree or higher	719	494 (68.71)	225 (31.29)			446 (62.03)	273 (37.97)			569 (79.14)	150 (20.86)		
**Years of service**				12.562	0.002			10.73	0.005			16.008	<0.001
<5	262	177 (67.56)	85 (32.44)			163 (62.21)	99 (37.79)			206 (78.63)	56 (21.37)		
5–15	344	230 (66.86)	114 (33.14)			207 (60.17)	137 (39.83)			263 (76.45)	81 (23.55)		
>15	306	240 (78.43)	66 (21.57)			220 (71.90)	86 (28.10)			270 (88.24)	36 (11.76)		
**Occupational injury status**				7.844	0.005			11.651	0.001			12.496	<0.001
No	740	540 (72.97)	200 (27.03)			498 (67.30)	242 (32.70)			616 (83.24)	124 (16.76)		
Yes	172	107 (62.21)	65 (37.79)			92 (53.49)	80 (46.51)			123 (71.51)	49 (28.49)		
**Geographical mobility**				7.855	0.005			8.695	0.003			7.478	0.006
No	65	56 (86.15)	9 (13.85)			53 (81.54)	12 (18.46)			61 (93.85)	4 (6.15)		
Yes	847	591 (69.78)	256 (30.22)			537 (63.40)	310 (36.60)			678 (80.05)	169 (19.95)		
**Physical health status**				43.884	<0.001			68.79	<0.001			28.45	<0.001
Good	513	405 (78.95)	108 (21.05)			383 (74.66)	130 (25.34)			442 (86.16)	71 (13.84)		
Fair	353	222 (62.89)	131 (37.11)			196 (55.52)	157 (44.48)			270 (76.49)	83 (23.51)		
Poor	46	20 (43.48)	26 (56.52)			11 (23.91)	35 (76.09)			27 (58.70)	19 (41.30)		
**COVID-19 infection status**				1.314	0.252			4.33	0.037			1.734	0.188
No	169	126 (74.56)	43 (25.44)			121 (71.60)	48 (28.40)			143 (84.62)	26 (15.38)		
Yes	743	521 (70.12)	222 (29.88)			469 (63.12)	274 (36.88)			596 (80.22)	147 (19.78)		
**Impact of COVID-19 pandemic**				25.889	<0.001			42.81	<0.001			23.73	<0.001
None	305	243 (79.67)	62 (20.33)			239 (78.4)	66 (21.6)			268 (87.9)	37 (12.1)		
Mild	552	377 (68.30)	175 (31.70)			327 (59.2)	225 (40.8)			437 (79.2)	115 (20.8)		
Severe	55	27 (49.09)	28 (50.91)			24 (43.6)	31 (56.4)			34 (61.8)	21 (38.2)		
**Insomnia**				171.957	<0.001			155.405	<0.001			100.447	<0.001
No	481	431 (89.60)	50 (10.40)			401 (83.37)	80 (16.63)			449 (93.35)	32 (6.65)		
Yes	431	216 (50.12)	215 (49.88)			189 (43.85)	242 (56.15)			290 (67.29)	141 (32.71)		
**Alcohol use disorder**				8.949	0.003			17.613	<0.001			18.713	<0.001
Male	271	211 (77.86)	60 (22.14)			203 (74.91)	68 (25.09)			243 (89.67)	28 (10.33)		
Female	641	436 (68.02)	205 (31.98)			387 (60.37)	254 (39.63)			496 (77.38)	145 (22.62)		

### Correlation analysis of variables

According to the correlation analysis, there was a significant positive correlation between anxiety, stress, and depression (*P* < 0.001). Alcohol dependence was positively correlated with anxiety (*P* < 0.001) and depression (*P* < 0.001). The insomnia index was significantly and positively correlated with stress (*P* < 0.001), anxiety (*P* < 0.001), depression (*P* < 0.001), and alcohol dependence (*P* < 0.001). Work-family conflict was positively correlated with stress (*P* < 0.001), anxiety (*P* < 0.001), and depression (*P* < 0.001). Leadership support is negatively correlated with stress (*P* < 0.001), anxiety (*P* < 0.001), and depression (*P* < 0.001). Workplace exclusion was positively correlated with stress, anxiety, and depression (*P* < 0.001). Proactive personality was negatively correlated with stress, anxiety, depression, and workplace rejection (*P* < 0.001) and positively correlated with leadership support (*P* < 0.001) (**Table 3**).

**Table 3:** Correlation of variables

	Mean	Standard deviation	1	2	3	4	5	6	7	8	9
**1. Stress**	8.67	7.80	1.00								
**2. Anxiety**	6.31	6.42	0.81†	1.00							
**3. Depression**	6.09	6.84	0.82†	0.78†	1.00						
**4. Alcohol dependence**	5.60	6.31	0.45†	0.49†	0.39†	1.00					
**5. Insomnia index**	7.36	4.89	0.59†	0.55†	0.55†	0.35†	1.00				
**6. Work family conflict**	4.58	1.69	0.38†	0.33†	0.34†	0.31†	0.31†	1.00			
**7. Leadership support**	4.81	1.46	−0.23†	−0.26†	−0.30†	−0.08†	−0.21†	−0.02	1.00		
**8. Workplace exclusion**	2.22	0.73	0.41†	0.39†	0.44†	0.23†	0.28†	0.30†	−0.30†	1.00	
**9. Proactive personality**	4.58	1.12	−0.20†	−0.19†	−0.26†	−0.04	−0.14†	−0.01	0.43†	−0.22†	1.00

*Significance level of 0.1%.

†Significance level of 5%.

### Multivariate logistic regression analysis

#### Risk factors for depression

The regression analysis showed that education positively predicted the level of depression (*P* = 0.036). The presence of work-related injuries at a construction site positively predicted the level of depression (*P* = 0.009). Physical health positively predicted depression (*P* = 0.014). Work-family conflict positively predicted depression levels (*P* = 0.002). Leadership support negatively predicted depression (*P* = 0.015). Workplace exclusion positively predicted depression (*P* < 0.001). An active personality negatively predicted depression levels (*P* < 0.001). Alcohol dependence positively predicted depression (*P* < 0.001). The insomnia index positively predicted depression levels (*P* < 0.001) (Table S1 in the **Online Supplementary Document**).

#### Risk factors of anxiety

Regression analysis showed that physical health problems positively predicted anxiety levels (*P* = 0.001). Leadership support negatively predicted anxiety levels (*P* < 0.05). Workplace exclusion positively predicted anxiety levels (*P* = 0.010). Alcohol dependence positively predicted anxiety levels (*P* < 0.001). The insomnia index positively predicted anxiety levels (*P* < 0.001) (Table S2 in the **Online Supplementary Document**).

#### Risk factors of pressure level

Physical health problems positively predicted stress levels (*P* = 0.036). Work-family conflict positively predicted stress levels (*P* < 0.001). Workplace exclusion positively predicted stress levels (*P* < 0.001). A proactive personality negatively predicted stress levels (*P* = 0.010). Alcohol dependence positively predicted stress levels (*P* < 0.001). The insomnia index positively predicted stress levels (*P* < 0.001) (Table S3 in the **Online Supplementary Document**).

## DISCUSSION

We observed that depression, anxiety, and stress levels were significantly associated with age, education, work-related injuries, COVID-19 status, insomnia, alcohol dependence, workplace exclusion, and work-family conflict among construction workers. Education level, work injuries, and physical health functioned as predictors of mental health outcomes. Work-family conflict, workplace exclusion, alcohol dependence, and insomnia were positively associated with depression, while proactive personality and leadership support were negatively associated with depression. Similarly, physical health, workplace exclusion, alcohol dependence, and insomnia were positively associated with anxiety. Additionally, having a proactive personality negatively influenced depression. Anxiety positively predicted physical health issues, workplace exclusion, alcohol dependence, and insomnia, whereas leadership support reduced anxiety levels.

Our study showed significant differences in the mental health of construction workers in terms of their demographics. The levels of depression, anxiety, and stress in individuals <35 years of age were significantly higher than those aged ≥35 years, reflecting the difference in stress across life stages. Higher education was significantly associated with depression, anxiety, and stress, reflecting increased social expectations and competitive pressures, which could be attributed to social responsibility. First, in China, people aged 25–35 years are traditionally expected to form new families, requiring them to fulfil the responsibilities of their parents or spouses. They simultaneously have to care for and support their parents. Aside from this, Chinese society generally regards senior intellectuals aged <35 years as the backbone of the country’s social progress and development, and demands they spend more time and energy to respond to the expectations of all sectors of society. Faced with these familial and societal pressures, this group is also at higher risk of mental health issues. We found that people with high educational qualifications in the construction industry are more affected by COVID-19 and are more prone to mental health problems than those with low educational qualifications. This might be because people with higher educational attainment have a greater awareness of the dangers of COVID-19, and thus have a greater impact on mental health.

We found that the insomnia index can positively predict the level of stress, anxiety, and depression [27]. Construction workers face pressure from multiple factors such as high-intensity physical labour, high-pressure working environments, and tight construction periods [28]. Therefore, improving their sleep quality and relieving stress are important for maintaining physical and mental health [29]. Relevant organisations (*e.g.* the International Labour Organization) should actively monitor the mental health of construction workers through appropriate management mechanisms to help them mitigate psychological stress and create a good internal interpersonal relationship, and create a more supportive work environment [30].

We observed that alcohol dependence positively predicted levels of depression, anxiety, and stress. This finding aligns with those of Jacob and colleagues [31]. The tense social environment during the COVID-19 pandemic aggravated alcohol dependence in some populations [4]. Another study found that patients with alcohol dependence had a higher risk of depression during the pandemic, which is the most common complication of mental illness in this population [32]. Alcohol dependence and depression appear to share some behavioural, genetic, and environmental risk factors, though they remain poorly understood [27]. Therefore, encouraging workers to avoid alcohol dependence through alternative coping strategies, such as music therapy and effective communication, may improve the mental well-being of construction workers.

Our data showed that leadership support negatively predicted depression and anxiety levels, while workplace exclusion positively predicted depression, anxiety, and stress levels. Song and colleagues [33] found that through internal reform and management mechanisms, and appropriate delegation of power to subordinates, workers' subjective feelings of leadership support can be improved, and a good working environment and interpersonal relationships can be created to alleviate their anxiety and pressure [33,34]. Other researchers have shown that social support can regulate and alleviate the impact of stress on psychological symptoms and improve individuals’ ability to adapt to their living environment [35], as well as alleviate the pressure and burden they feel, and help individuals be less affected by symptoms of depression and anxiety [36]. In our study, work-family conflict positively predicted the level of depression and stress, and leadership support negatively predicted the level of depression and anxiety.

Leadership support was negatively correlated with workplace exclusion in our sample, and positively correlated with proactive personality. This indicates that a good employment relationship can improve the anxiety, depression, and stress levels of construction workers. Studies have found that leaders, whether intentionally/directly or unintentionally/indirectly, can promote or inhibit the process of exclusion [37]. Proactive people are more likely to carefully design their work and strive to create favourable working conditions [38], especially as they are more likely to successfully build good employment relationships and improve their mental health [39]. To address these challenges, open communication channel should be created so that workers can share their problems and concerns. Satisfying their psychological needs can promote their creative performance and mental health, which can be achieved by improving employee relations and developing organisational culture [40–42].

This study has some limitations. We used online questionnaire to collect data, which may have resulted in information loss or response bias due to response bias [43]. Additionally, we adopted a cross-sectional design for this study, meaning that we collected data at a single time point, prohibiting us from observing changes that may occur over time or establishing causal relationships. In the future, we aim to conduct a cohort study to better examine the changes over time in employees' mental health. Lastly, we used a self-assessment scale to assess psychological states such as depression, anxiety, and stress. Since these responses are influenced by participants' subjective feelings and memory recall, there is a possibility of bias self-reporting and recall bias [44].

## CONCLUSIONS

In this cross-sectional study, we found notable differences in anxiety, depression, and stress levels among from different types of construction workers, and identified alcohol dependence and work-related stress as central contributors to their mental health challenges. Our findings underscore the need for targeted interventions such as improving workplace safety and ergonomics, enhancing leadership-driven well-being initiatives, and strengthening peer support networks to address these stressors and foster a more supportive occupational environment. Practically, the study offers actionable insights for stakeholders to prioritise measures that reduce injury risks, enhance workers’ sense of security through policy adjustments and resource access, and integrate mental health support into organisational practices. It also highlights opportunities for future inquiry, including investigating the long-term effects of such interventions on workers’ psychological and occupational outcomes, exploring how socioeconomic factors like migration status or job insecurity intersect with workplace conditions to influence mental health, and comparing findings across high-risk industries to develop transferable frameworks for promoting mental health in precarious employment settings.

## Additional material


Online Supplementary Document

## References

[1] Liang Q Zhou Z Ye G Shen L Unveiling the mechanism of construction workers’ unsafe behaviors from an occupational stress perspective: A qualitative and quantitative examination of a stress–cognition–safety model. Saf Sci. 2022;145:105486. 10.1016/j.ssci.2021.105486

[2] Nwaogu JM Chan APC Work-related stress, psychophysiological strain, and recovery among on-site construction personnel. Autom Construct. 2021;125:103629. 10.1016/j.autcon.2021.103629

[3] Williams JM Andersen MB Psychosocial antecedents of sport injury: Review and critique of the stress and injury model. J Appl Sport Psychol. 1998;10:5–25. 10.1080/10413209808406375

[4] Garnett C Jackson S Oldham M Brown J Steptoe A Fancourt D Factors associated with drinking behaviour during COVID-19 social distancing and lockdown among adults in the UK. Drug Alcohol Depend. 2021;219:108461. 10.1016/j.drugalcdep.2020.108461 33454159 PMC7807168

[5] Bakker AB Tims M Derks D Proactive personality and job performance: The role of job crafting and work engagement. Hum Relat. 2012;65:1359–78. 10.1177/0018726712453471

[6] Fu X Guo X Lu J Zhou W Lu Y Acceptance of human papillomavirus vaccine among boys in Asia: A narrative review. Hum Vaccin Immunother. 2024;20:2429894. 10.1080/21645515.2024.2429894 39611606 PMC11610555

[7] Ferris DL Brown DJ Berry W Lian H The development and validation of the Workplace Ostracism Scale. J Appl Psychol. 2008;93:1348–66. 10.1037/a0012743 19025252

[8] Xue W Maozhong L Ming L Qin L Lu K Hui X Mycoplasma Pneumoniae Triggers Pneumonia Epidemic in Autumn and Winter in Beijing: A Multicenter, Population-based Epidemiological Study between 2015 and 2020. Emerg Microbes Infect. 2022;11:21–9. 10.1080/22221751.2022.2078228 PMC9176688 35582916

[9] Jin Y Wang Y An J The impact of employee perceived stress on sleep quality: The moderating role of mindfulness and the mediating role of anxiety. Psychol Sci. 2022;45:433–8.

[10] Seibokaite L Endriulaitiene A The role of personality traits, work motivation and organizational safety climate in risky occupational performance of professional drivers. Balt J Manag. 2012;7:103–18. 10.1108/17465261211195892

[11] Liang Q Leung M Amed K How adoption of coping behaviors determines construction workers’ safety: A quantitative and qualitative investigation. Saf Sci. 2021;133:105035. 10.1016/j.ssci.2020.105035

[12] Alzaffin K Kaye S Watson A Haque MM A Data Fusion Approach of Police-Hospital Linked Data to Examine Injury Severity of Motor Vehicle Crashes. Accid Anal Prev. 2023;179:106897. 10.1016/j.aap.2022.106897 36434986

[13] Bae Y Woo S Study of Satisfaction Levels on Construction Engineering Education in Cyber University and Its Improvements. KSCE J Civ Eng. 2019;23:499–506. 10.1007/s12205-018-1552-y

[14] Lim S Chi S Lee JD Lee HJ Choi H Analyzing psychological conditions of field-workers in the construction industry. Int J Occup Environ Health. 2017;23:261–81. 10.1080/10773525.2018.1474419 29989485 PMC6147106

[15] Hasan MK Mahmud M Habib AKMA Motakabber SMA Islam S Review of Electric Vehicle Energy Storage and Management System: Standards, Issues, and Challenges. J Energy Storage. 2021;41:102940. 10.1016/j.est.2021.102940

[16] Schecke H Bohn A Scherbaum N Mette C Alcohol use during COVID-19 pandemic on the long run: findings from a longitudinal study in Germany. BMC Psychol. 2022;10:266. 10.1186/s40359-022-00965-8 36376933 PMC9661459

[17] Herbener AB Damholdt MF Are lonely youngsters turning to chatbots for companionship? The relationship between chatbot usage and social connectedness in Danish high-school students. Int J Hum Comput Stud. 2025;196:103409. 10.1016/j.ijhcs.2024.103409

[18] Thomas PA Teas E Friedman E Barnes LL Rolston MRS Ferraro KF Early-Life Parental Affection, Social Relationships in Adulthood, and Later-Life Cognitive Function. J Aging Health. 2024:8982643241303589. 10.1177/08982643241303589 39629987 PMC12134153

[19] Elahi NS Abid G Contreras F Fernández IA Work-family and family-work conflict and stress in times of COVID-19. Front Psychol. 2022;13:951149. 10.3389/fpsyg.2022.951149 36304883 PMC9595337

[20] Kayaalp A Page KJ Rospenda KM Caregiver Burden, Work-Family Conflict, Family-Work Conflict, and Mental Health of Caregivers: A Mediational Longitudinal Study. Work Stress. 2021;35:217-40. 10.1080/02678373.2020.1832609 34483432 PMC8412180

[21] Cao CH Psychometric evaluation of the depression, anxiety, and stress scale-21 (DASS-21) among Chinese primary and middle school teachers. BMC Psychol. 2023;11:209. 10.1186/s40359-023-01242-y 37452365 PMC10349442

[22] Morin CM Belleville G Bélanger L Ivers H The Insomnia Severity Index: psychometric indicators to detect insomnia cases and evaluate treatment response. Sleep. 2011;34:601-8. 10.1093/sleep/34.5.601 21532953 PMC3079939

[23] Lan YM Li CP Wang JY Meng X The benefits and costs of employee boundary-spanning behavior: Evidence from a meta-analysis. Acta Psychol Sin. 2022;54:665–83. 10.3724/SP.J.1041.2022.00665

[24] Cheng B Coates JM Gullo MJ Feeney GFX Kavanagh DJ Young RM Relationship between alcohol craving dimensions and features of comorbid mental health in an alcohol dependent sample. Addict Behav. 2022;124:107106. 10.1016/j.addbeh.2021.107106 34530206

[25] Zhang Q. [Research on the Influence Mechanism of Risk Propensity on Hazard Perception of New Generation Construction Workers] [Master's Thesis]. Jiangsu, China: China University of Mining and Technology; 2023. Chinese.

[26] Bateman TS Crant JM The proactive component of organizational behavior: A measure and correlates. J Organ Behav. 1993;14:103–18. 10.1002/job.4030140202

[27] Wang ZH Zhang S Zhu Y Zhang ZY Research on the Work Pressure Status of Project Management Personnel in China—Based on the Perspective of the Entire Project Implementation Process. Project Management Technology. 2022;20:14–21.

[28] Noblet A Rodwell J McWilliams J Organizational change in the public sector: Augmenting the demand control model to predict employee outcomes under New Public Management. Work Stress. 2006;20:335–52. 10.1080/02678370601050648

[29] Oni OZ Olanrewaju A Khor SC Akinbile BF A comparative analysis of construction workers’ mental health before and during COVID-19 pandemic in Nigeria. Frontiers in Engineering and Built Environment. 2023;3:63–75. 10.1108/FEBE-05-2022-0018

[30] Jiang P Zhang LH Can Weiqu lead to compromise? The impact of workplace ostracism on individual performance from a self-presentation perspective. Acta Psychol (Amst). 2021;53:400–12. 10.3724/SP.J.1041.2021.00400

[31] Kristensen K, Eskildsen J. Design of PLS-Based Satisfaction Studies. In: Vinzi VE, Chin WW, Hensler J, Wang G, editors. Handbook of Partial Least Squares: Concepts, Methods and Applications. Berlin, Germany: Springer Nature; 2010. p 247–277.

[32] Zegel M Rogers AH Vujanovic AA Zvolensky MJ Alcohol use problems and opioid misuse and dependence among adults with chronic pain: The role of distress tolerance. Psychol Addict Behav. 2021;35:42–51. 10.1037/adb0000587 32364397

[33] Song K Zhao Z Saha A Kundu J Receiving financial support and its association with late-age depression: The mediating role of social engagement. Exp Gerontol. 2025;199:112647. 10.1016/j.exger.2024.112647 39643252

[34] Jessee L Bordone V Hank K Adult intergenerational proximity and parents’ depressive symptoms: A bidirectional approach. Soc Sci Res. 2025;125:103094. 10.1016/j.ssresearch.2024.103094 39615957

[35] Kwon M Livingston JA Wang W Hequembourg AL Longitudinal association between adolescent sexual identity and sleep quality: The mediating roles of peer victimization and perceived social support. Sleep Health. 2025;11:25–32. 10.1016/j.sleh.2024.09.012 39523194 PMC11805634

[36] Zheng X, Huang Y, Li Y. Investigation and Analysis of the Mental Health Status of Bank Employees in a City. Times Finance. Available: https://jogh.org/. Accessed: 25 April 2025.

[37] Jung DY Yoon KH Jo MY Jeong HJ Kweon YS Depression, Anxiety and Associated Factors Among Korean Adolescent Students During COVID-19. J Korean Acad Child Adolesc Psychiatry. 2024;35:230–42. 10.5765/jkacap.220039 39380563 PMC11456651

[38] Lin YK Saragih ID Lin CJ Liu HL Chen CW Yeh YS Global prevalence of anxiety and depression among medical students during the COVID-19 pandemic: a systematic review and meta-analysis. BMC Psychol. 2024;12:338. 10.1186/s40359-024-01838-y 38858700 PMC11163725

[39] Bangalan S Agnes MC A pilot study of “AKBAY” self-help intervention for university students with moderate symptoms of depression and anxiety. J Public Ment Health. 2024;23:279–88. 10.1108/JPMH-04-2024-0045

[40] Lim S Chi S Lee JD Lee HJ Choi H Analyzing psychological conditions of field-workers in the construction industry. Int J Occup Environ Health. 2017;23:261–81. 10.1080/10773525.2018.1474419 29989485 PMC6147106

[41] Nwaogu JM Chan APC Evaluation of multi-level intervention strategies for a psychologically healthy construction workplace in Nigeria. Journal of Engineering, Design and Technology. 2021;19:509–36. 10.1108/JEDT-05-2020-0159

[42] Syed A Syed SA Khan M Frequency of depression, anxiety and stress among the undergraduate physiotherapy students. Pak J Med Sci. 2018;34:468–71. 10.12669/pjms.342.12298 29805428 PMC5954399

[43] Johnson C Dupuis JB Robichaud W Pone EK LeBlanc CP Weight gain and social support networks in Canadian federal correctional facilities. Int J Prison Health. 2024;20:466–78. 10.1108/IJOPH-08-2023-0053

[44] Jiao H Wong IP Lin Z Understanding customer multi-interactions, trust, social support and voluntary performance in smart restaurants. J Hosp Tour Technol. 2024;15:717–36. 10.1108/JHTT-11-2023-0384

