# Supplementary material for: Construction workers’ depression, anxiety, stress, and risk factors in China: a cross-sectional study
Source: J Glob Health. 2025 Jul 21;15:04167. doi: 10.7189/jogh.15.04167 (PMC12278889; doi:10.7189/jogh.15.04167)
Supplement: Online Supplementary Document [file jogh-15-04167-s001.pdf]

Supplement to: Ling Z, Zheng K, Yu J, Xu Y, Zhang M, Liu Y, Wang J, Sheng Y, Liu X, Huang L. Construction workers' depression, anxiety, stress, and risk factors in China: a cross-sectional study. J Glob Health 2025;15:04167.

Table S1. Logistic regression analysis of depression

Variable	*B*	*SE*	*β*	*t*	*p*	95%*CI*
**(constant)**	-1.84	1.96		-0.94	0.348	[-5.692,2.010]
Education	0.68	0.33	0.06	2.10	0.036	[0.045,1.321]
Whether there is work-related injury	1.15	0.44	0.07	2.60	0.009	[0.282,2.010]
Physical health	0.79	0.32	0.07	2.46	0.014	[0.160,1.415]
Work family conflict	0.35	0.12	0.09	3.05	0.002	[0.126,0.579]
leadership support	-0.33	0.14	-0.07	-2.44	0.015	[-0.592,0.064]
Workplace exclusion	2.04	0.26	0.21	7.73	0.000	[1.519,2.554]
Proactive personality	-0.73	0.17	-0.12	-4.30	0.000	[-1.061,0.396]
Alcohol dependence	0.15	0.03	0.14	5.05	0.000	[0.094,0.214]
Insomnia index	0.51	0.04	0.36	12.68	0.000	[0.433-0.591]

Table S2. Logistic regression analysis of anxiety

Variable	*B*	*SE*	*β*	*t*	*p*	95%*CI*
**(constant)**	-1.28	1.81		-0.70	0.481	[-4.832,2.28]
Physical health status	1.00	0.30	0.09	3.37	0.001	[0.417,1.575]
leadership support	-0.32	0.12	-0.07	-2.59	0.010	[-0.565,0.077]
Workplace exclusion	1.39	0.24	0.16	5.70	0.000	[0.910,1.865]
Alcohol dependence	0.28	0.03	0.27	9.86	0.000	[0.222,0.333]
Insomnia index	0.44	0.04	0.33	11.75	0.000	[0.365,0.512]

Table S3. Logistic regression analysis of pressure level

Variable	*B*	*SE*	*β*	*t*	*p*	95%*CI*
**(constant)**	-1.35	2.16		-0.63	0.532	[-5.582,2.884]
Physical health	0.74	0.35	0.06	2.10	0.036	[0.049,1.428]
Work family conflict	0.53	0.13	0.11	4.15	0.000	[0.278,0.777]
Workplace exclusion	1.83	0.29	0.17	6.32	0.000	[1.263,2.401]
Proactive personality	-0.48	0.19	-0.07	-2.58	0.010	[-0.845,0. 114]
Alcohol dependence	0.24	0.03	0.20	7.27	0.000	[0.178,0.309]
Insomnia index	0.63	0.04	0.40	14.24	0.000	[0.545,0.719]
